# Polymorphisms in human immunoglobulin heavy chain variable genes and their upstream regions

**DOI:** 10.1093/nar/gkaa310

**Published:** 2020-05-04

**Authors:** Ivana Mikocziova, Moriah Gidoni, Ida Lindeman, Ayelet Peres, Omri Snir, Gur Yaari, Ludvig M Sollid

**Affiliations:** 1 K.G.Jebsen Centre for Coeliac Disease Research and Department of Immunology, University of Oslo and Oslo University Hospital, 0372 Oslo, Norway; 2 Faculty of Engineering, Bar Ilan University, Ramat Gan 5290002, Israel

## Abstract

Germline variations in immunoglobulin genes influence the repertoire of B cell receptors and antibodies, and such polymorphisms may impact disease susceptibility. However, the knowledge of the genomic variation of the immunoglobulin loci is scarce. Here, we report 25 potential novel germline IGHV alleles as inferred from rearranged naïve B cell cDNA repertoires of 98 individuals. Thirteen novel alleles were selected for validation, out of which ten were successfully confirmed by targeted amplification and Sanger sequencing of non-B cell DNA. Moreover, we detected a high degree of variability upstream of the V-REGION in the 5′UTR, L-PART1 and L-PART2 sequences, and found that identical V-REGION alleles can differ in upstream sequences. Thus, we have identified a large genetic variation not only in the V-REGION but also in the upstream sequences of IGHV genes. Our findings provide a new perspective for annotating immunoglobulin repertoire sequencing data.

## INTRODUCTION

Immunoglobulins are an important part of the adaptive immune system. They exert their function either as the antigen receptor of B cells that is essential for the antigen presentation capacity of these cells (1), or as secreted antibodies that survey extracellular fluids of the body. Immunoglobulins can bind a plethora of antigen epitopes via their paratopes, which are composed of combinations of heavy and light chain variable regions. A huge diversity of paratopes is established by recombination of variable (V), diversity (D) (not in light chains) and joining (J) genes, and the pairing of heavy and light chains (2). The genes of the heavy chain are located on chromosome 14 (14q32.33) (3), while the light chain genes are present on two separate loci, kappa and lambda, which are located on chromosome 2 (2p11.2) and chromosome 22 (22q11.2) respectively (4).

These loci remain incompletely characterized due to the fact that they contain many repetitive sequence segments with many duplicated genes (5), which makes it difficult to correctly assemble short reads from whole genome sequencing. To this date, a limited number of genomically sequenced (6–8) and inferred (9,10) haplotypes of the heavy chain and the two light chain loci have been described. Different databases exist for genomic immune receptor DNA sequences (IMGT/GENE-DB (11)), putative novel variants from inferred data (IgPdb, https://cgi.cse.unsw.edu.au/∼ihmmune/IgPdb/information.php) or entire immune receptor repertoires (OGRDB (12)).

The usage of immunoglobulin heavy chain variable (IGHV) genes and their mutational status are most frequently studied in relation to cancer (13,14), responses to vaccines (15,16), or in autoimmune diseases (17–19). Most IGHV genes have several allelic variants and more alleles are being discovered as a result of adaptive immune receptor repertoire-sequencing (AIRR-seq) (20,21). Software tools such as TIgGER (22,23), IgDiscover (24) and partis (25) allow to infer germline alleles from such repertoire data. Based on these inferred alleles, the data can then be input to other tools that infer haplotypes and repertoire deletions (26). Incorrect annotation could possibly lead to inferring wrong deletions and biased assessments. Therefore, having a full overview of germline variants is essential for studying the adaptive immune response with high accuracy.

Some allelic variants have been associated with increased disease susceptibility (27,28), yet the impact of immunoglobulin gene variation on disease risks is still unknown (29). These regions have not been sufficiently covered in the numerous genome wide association studies performed to date. More comprehensive maps of polymorphisms are required for proper analysis.

Here, we have used previously generated AIRR-seq data (30) from naïve B cells of 98 Norwegian individuals to identify novel IGHV alleles, a selection of which we then validated from genomic DNA (gDNA) of non-B cells, i.e. T cells and monocytes. We also analyzed the sequences upstream of the V-REGION, and constructed consensus sequences for the upstream variants present in the cohort. These results expand our knowledge of this important locus and deepen our understanding of allelic diversity within the Caucasian population. In addition, the result of this study can be used to improve the accuracy of currently used bioinformatics tools for the analysis of immunoglobulin repertoire sequencing data.

## MATERIALS AND METHODS

### AIRR sequencing of naïve B cells

The data was obtained as a part of a previously published study (30) and is available in the European Nucleotide Archive (ENA) under the accession number PRJEB26509. In summary, naïve B cells from 100 individuals were sorted from peripheral blood mononuclear cells (PBMCs). The RNA was isolated and quality checked before being sent to AbVitro, Inc for library preparation and sequencing on Illumina MiSeq (2 × 300 bp). About half of the cohort are celiac disease patients, and these subjects were included to increase the diversity of the cohort. Of note, this study was not designed and powered to perform comparative analysis of allelic frequencies between patients and controls.

### Amplification of target genomic regions

Genomic DNA (gDNA) was isolated from previously sorted T cells and monocytes (CD19^−^ CD3^+^/CD14^+^) (30) using the QiaAmp DNA mini kit (Qiagen), and the concentration was measured on Nanodrop.

Primers for validation were designed by PrimerBLAST using the reference genome as a template. The nucleotide sequences of primers with additional details can be found in the Supplementary material. For amplification of genes IGHV3-7, IGHV3-20, and IGHV3-21, primers from a recently-published study (31) were used. All oligos were synthesized and purified (RP-cartridge) by Eurogentec.

The target regions of the gDNA were amplified by touch-down PCR using Q5^®^ Hot Start High-Fidelity DNA Polymerase (NEB). Approx. 100–200 ng gDNA from an individual with a suspected polymorphism was used as a template. The PCR started with two cycles with the annealing temperature of 70°C. The touch-down part of the PCR consisted of 10 cycles with the annealing temperature decreasing from 70°C to 60°C by 1°C every cycle. In the next 13 cycles, the annealing temperature remained constantly at 60°C, and the last step of the PCR was the final extension at 72°C. The length of the PCR product varied depending on the amplified gene, ranging between 750 and 986 bp.

### Cloning

The PCR products were cleaned using the Monarch^®^ DNA Gel Extraction Kit (NEB), and 3′ end A-overhangs were added by NEBNext^®^ dA-Tailing Module (NEB). The A-tailed products were subsequently cloned into pGEM^®^-T Easy vector (Promega) using the manufacturer's protocol. For transformation, 4 μl of the ligation reaction were used to transform 90 μl XL10 CaCl_2_-competent cells. After transformation, 100 μl cells were plated on LB_amp_ 50 μg/ml plates that have been previously coated with IPTG/X-Gal (40 μl 100 mM IPTG + 16 μl 50 mg/ml X-Gal). The IPTG/X-Gal treatment allows for selection of successfully transformed colonies based on color. After overnight incubation at 37°C, white colonies were picked and the plasmids were isolated using the Monarch^®^ Plasmid Miniprep Kit (NEB). To verify that the picked colonies contain an insert of the correct size, a PCR was performed using the same primers as for the amplification of gDNA, and the products were analyzed by gel electrophoresis (1% agarose, 100 V, 35 min). The size of the PCR product was between 750 and 986 bp, depending on the gene amplified.

### Sanger sequencing

Sanger sequencing of the plasmid DNA containing the correct-sized insert, or direct sequencing of a pure single-gene PCR product (IGHV3–9*01_T307C), was performed by Eurofins. The resulting sequences were trimmed to remove the vector and primer sequences. V-gene annotation was done by IMGT/HighV-QUEST (32). To check for polymorphisms in the introns, leader regions and 5′UTRs, the trimmed sequences were aligned by MUSCLE (33,34) to the reference alleles of the amplified gene, where available, and checked for polymorphisms. Alignments were visually inspected in Jalview (35) and/or UGENE (36).

The sequences were named based on the amplified gene, followed by the closest reference allele and the V-REGION polymorphism, which was determined by IMGT/V-Quest (37). In cases of ambiguous annotation, such as for IGHV3-64*05/IGHV3-64D*06, the sequence was annotated manually based on the alignment with focus on the differences in the upstream sequences. The gDNA sequences of validated novel alleles were submitted to GenBank and the accession numbers were subsequently sent to IMGT. As of now, seven of the submitted alleles have been added to the IMGT/GENE-DB and were given the following allele names: MN337615 (IGHV1-2*02_G207T) was named IGHV1-2*07; MN337616 (IGHV1-3*01_T35A) was named IGHV1-3*05; MN337618 (IGHV1-69*01_C243T) was named IGHV1-69*19; MN337619 (IGHV3-7*03_G144A) was named IGHV3-7*05; MN337623 (IGHV3–21*01_C255T) was named IGHV3-21*06; MN337624 (IGHV3-64D*06_G258T) was named IGHV3-64D*08; and MN337625 (IGHV3-64D*06_C210A) was named IGHV3-64D*09.

### AIRR-seq data pre-processing

The AIRR-seq data was pre-processed as described originally (30) using pRESTO (38). Two individuals were excluded from the analysis due to low sequencing depth (<2000). The excluded samples were S98 (Accession ERS2445863) and S96 (Accession ERS2445861).

### Novel allele discovery and genotype inference

The pre-processed sequences were annotated by IgBLAST 1.14.0 (39) with modified parameters, and the IMGT germline database (24) from January 2019 was used as a reference. We experienced that the default settings resulted in incorrect annotation for some genes. This was particularly obvious for the allele IGHV5-51*03, which was incorrectly annotated as IGHV5-51*01 with one mutation C45G, corresponding to the already known allele *03. These two alleles differ only by one nucleotide, and it was the length of the reference allele that seemed to affect whether or not the sequence was correctly annotated by IgBLAST. The reference for *03 is 2 nt shorter than the reference sequence for *01, while sequences in our data corresponding to IGHV5-51*03 were matching the length of allele *01. Adjusting the IgBLAST parameters reward to 0 and penalty to –3 resolved this annotation problem. These parameters were also induced manually in the IgDiscover alignment step.

Genotype inference and novel allele discovery was performed by TIgGER v0.3.1 and IgDiscover v0.11. The reason for using two software suites was to increase our confidence in the allele inference. TIgGER and IgDiscover both utilize the output of aligner tools, which makes the output data more comparable. For novel allele detection we tested the parameters of the TIgGER function ‘findNovelAlleles’: (i) germline_min 50, 100 and 200 (default); (ii) j_max 0.15 (default), 0.3 and 0.5; (iii) min_seqs 25 and 50 (default). Different parameters resulted in different sets of novel alleles identified (not shown). To allow for discovery of novel alleles in lowly-expressed genes, we set the germline_min parameter to 50. The rest of the parameters, including j_max and min_seqs, were left as default. The novel alleles were further submitted for genotype inference, using a Bayesian approach, for each individual. As for IgDiscover, the default parameters for novel allele and genotype calls were applied. All alleles were inferred only up to position 312 of the V-REGION (IMGT unique numbering scheme) .The results of alignment and genotype inference by TIgGER were processed using a similar pipeline to the one used in http://www.vdjbase.org (40) with slight modifications. Analysis of the IgDiscover and TIgGER output was performed in R Studio version 3.6.0.

### Filtering out false positive suspects

Errors that occur during the PCR reaction and/or sequencing could result in a false novel IGHV allele call. To filter out the suspected false positive signals, it was necessary to determine the mismatch frequency for all novel allele candidates (Supplementary Figure S1). First, for each gene with a suspected novel allele, we gathered all sequences that could be mapped to the closest germline V allele in a pre-realigned repertoire (prior to novel allele detection). Within these sequences, the position of the suspected polymorphism was inspected in all individuals in whom the novel allele candidate was found. A mismatch frequency was estimated as the fraction of sequences with the suspected polymorphism out of all sequences that were aligned to the V germline allele in that individual. For example, for novel allele IGHV3-21*01_A152G, in each individual carrying the suspected polymorphism, we collected all sequences that were mapped to IGHV3-21*01 in the pre-realigned repertoire and examined their position 152. Then, in this example, the mismatch frequency was estimated as the number of sequences whose position 152 did not match the germline ‘A’ nucleotide, divided by the number of total sequences aligned to IGHV3-21*01. Novel allele candidates with mismatch frequency lower than 0.35 in all carriers were considered as false positives. These included alleles with mutation patterns A152G, T154G, and A85C, namely IGHV3-11*01_A152G, IGHV3-21*01_A152G, IGHV3-21*01_A85C, IGHV3-30-3*01_A85C, IGHV3-33*01_A152G, IGHV3-33*01_T154G, IGHV3-48*01_A85C, IGHV3-9*01_A152G, and IGHV3-9*01_A85C. Two additional novel alleles were excluded (IGHV3-30*18_A85C and IGHV3-23*01_A85C) despite having mismatch frequency higher than 0.35. This was due to the fact that they appeared only in four individuals that were sequenced in a different batch separately from the 96 remaining samples, and they had 100% mutation frequency. On top of that, A to C mutation is the most common substitution error in Illumina MiSeq (41). In addition, IGHV3-23*01_A152G was also filtered out as a false positive since it had the same A to G polymorphism as the other excluded novel allele candidates.

### Analysis of gene and allele usage

Following the inference of genotype for each individual, we used IgBLAST 1.14.0 (39) to re-align each individual's sequences with their own personalized germline IGHV database as inferred by TIgGER. To compare the relative gene usage in individuals carrying different allele combinations, we selected sequences with V-REGION length >200 nt and up to three mutations. It was necessary that each sequence did not contain more than one allele in the V-REGION annotation (V_CALL). However, since duplicated genes can have shared alleles, this often results in ambiguous allele assignments, where the sequence's V_CALL annotation contains more than one gene. Yet, we aimed to keep such sequences, while removing the ambiguous allele assignment that occurred due to alignment issues rather than duplicated reference. Therefore, for the purpose of allele usage analysis we renamed the annotation of sequences with the V_CALL ‘IGHV3-23*01, IGHV3-23D*01’ and changed it to ‘IGHV3-23*01D’, indicating that the sequence can come from either of the genes. Similarly, the annotation ‘IGHV1-69*01, IGHV1-69D*01’ was changed to IGHV1-69*01D; and ‘IGHV2-70*04, IGHV2-70D*04’ was changed to IGHV2-70*04D. In addition, the reference sequences for IGHV3-30-5*01 and IGHV3-30*18 are also identical; and we therefore renamed such annotations to IGHV3-30X*doub; and IGHV3-30X*trip if the sequence annotation also contained IGHV3-30*01 as another possible assignment. Afterwards, all remaining sequences with multiple allele annotations in the V_CALL were filtered out. To visualize the relative gene usage, we first calculated the relative usage fraction of each allele separately. The relative usage fraction of an allele was determined by counting the sequences with identical V_CALL annotation and dividing that number by the sum of all sequences within an individual's IGHV repertoire. Next, we summed up the relative usage fractions of alleles belonging to the same gene that were present in the same individual. Finally, we plotted the relative usage of each gene across all individuals. All genes that were detected in at least one individual were included. The custom code for creating the gene usage plots can be found at https://github.com/ivanamik/IgPolym/blob/master/GeneUsagePlots.R.

### Inference of upstream sequences (5′UTR, L-PART1 and L-PART2)

**Figure 1.**
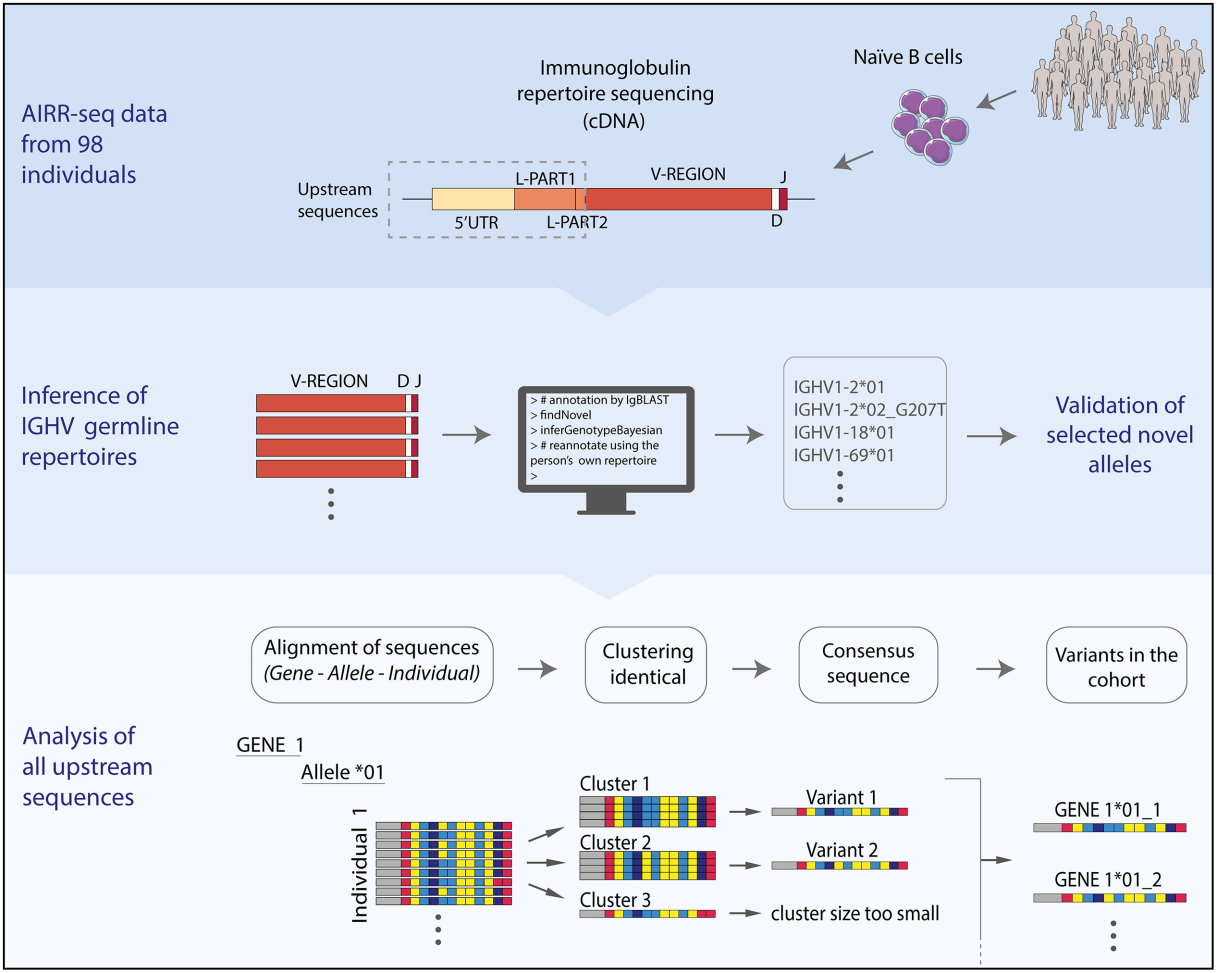
**Schematic representation of the data analysis**. In this study, we used material from a Norwegian cohort of 98 individuals (30). Following the initial preprocessing of the data, we inferred the germline V-gene repertoires of all individuals in the cohort and identified novel alleles using the software suites TIgGER and IgDiscover. The availability of genomic DNA of the same individuals allowed us to verify some of our findings from the analysis of the AIRR-seq data. Since the validation attempts revealed polymorphisms outside of the V-REGION, we decided to analyze the upstream sequences, i.e. 5′UTR, L-PART1 and L-PART2. We used a custom approach for this analysis based on clustering identical variants. More details about the protocols and analysis can be found in the methods section.

After the novel allele inference and genotyping, we analyzed the upstream sequence regions that consist of (5′-3′) 5′UTR, L-PART1, and L-PART2 (Figure 1). For this analysis, only sequences with up to three mutations in the V-REGION and a single assignment V_CALL were selected. The custom code for the analysis of the upstream sequences, as described below, is available at https://bitbucket.org/yaarilab/cluster_5utr/src/master/ .The following procedure was employed. First, the upstream sequences were extracted from the data by splitting them away at the start of the V-REGION. Despite removing the V-REGION and everything downstream of it, we kept the original V allele annotation. This means that each upstream sequence was annotated based on the original V-REGION to which it belonged. The upstream sequences were then aligned by the last (3′ end) nucleotide of L-PART2, which was to become position –1 (Figure 3). Next, due to slight variation in the 5′UTR length of sequences with the same allele annotation, it was necessary to trim the longest 5′UTRs, as this could interfere with the clustering. To determine where to trim the longer sequences, we filled the 5′ ends of the shorter sequences with Ns to match the length of the longest sequence. We then trimmed all sequences after the first position, at which 95% of sequences contained N. After that, we removed all artificially added Ns. Subsequently, we removed sequences with extremely short 5′UTRs. To do this, we estimated the frequency of sequence lengths, and the lengths with frequency above 0.1 were considered frequent. Sequences shorter than the shortest frequent sequence length were filtered out; and sequences longer than the longest frequent sequence length were trimmed to match its length. All remaining upstream sequences were used for clustering. By applying ClusterSets.py (--ident 0.999, --length 0.5) and BuildConsensus.py (--freq 0.6) from pRESTO, we constructed clusters that resulted in consensus sequences for each allele. There was no limitation regarding the number of clusters per allele. For each cluster we calculated its frequency based on the number of sequences assigned to it. Clusters with frequency below 0.1 or with <10 sequences were removed. For each allele, consensus sequences from all individuals were trimmed to match the shortest consensus sequence, and identical sequences were re-collapsed by allele and individual. In some of the consensus sequences, one of the nucleotides was marked with ambiguous assignment (N) by the BuildConsensus.py function. In such cases, the original cluster was split into two clusters based on the ambiguous assignment and consensus sequences were reconstructed manually. Finally, to create the consensus upstream sequences, for each allele the trimmed sequences were submitted to ClusterSets.py (--ident 1.0, --length 1.0) and BuildConsensus.py (--freq 0.6) functions and as a result, for each gene and allele a set of consensus V upstream sequences were gathered. In the last step, we compared and collapsed identical sequences from all individuals to create a database of upstream sequences in the cohort.

### Analysis of the reference germline upstream sequences

Reference germline sequences of the upstream sequences, including the 5′UTR, were obtained from the IMGT GENE-DB and by searching through the IMGT ‘Gene tables’ in order to get an alternative longer sequence if available. The reference upstream sequences longer than 150 nt were aligned using the MUSCLE tool at EMBL-EBI (34), and the alignment was visualized by Jalview (35) to look for conserved regions. The obtained consensus sequences of conserved regions were compared to IMGT resources for annotation. The TATA-boxes were determined based on either the reference annotation by IMGT, searching through previous studies, or by looking for a TA-rich region downstream of the octamer. Promoters previously described by older studies include that of IGHV6 (42) (with two TATA-boxes) and IGHV1 (43). The IGHV2 analysis was based on the available upstream reference sequences of IGHV2-5*01,*02 and IGHV2-70D*04,*14. IGHV3 schematic promoter representation was based on the upstream reference genomic sequences of IGHV3-43*01, IGHV3-48*02, IGHV3-49*03, IGHV3-64*02, IGHV3-64D*06 and the genomic sequences obtained by Sanger sequencing of IGHV3-7*02 and IGHV3-64D*06. The IGHV4 schematic representation of the promoter was based on the reference genomic sequences of IGHV4-4*07 and *08; IGHV4-28*01,*02,*07; IGHV4-30-2*06; IGHV4-30-4*07; IGHV4-31*02; IGHV4-34*01,*02,*11; IGHV4-38-2*02; IGHV4-39*01; IGHV4-59*01,*02,*11; and IGHV4-61*01,*08,*09.

## RESULTS

In this study, we used an AIRR-seq dataset from a cohort of 98 individuals (30) aiming to characterize novel IGHV alleles that might be present in the data, as well as analyze the sequences upstream of the V-REGION and create a table of previously unexplored upstream variants (Figure 1). To validate our inferences from the AIRR-seq data analysis, genomic DNA of the same individuals was isolated from non-B cells, i.e. T cells and monocytes. The reason for using non-B cells for validation was to avoid capturing sequences with somatic hypermutation that occurs in primed B cells.

We used two germline inference tools, TIgGER (22,23) and IgDiscover (24), to characterize novel alleles and to create a personalized germline reference of IGHV alleles for each individual (aka genotype). The purpose of using two different software tools was to increase our confidence in the inference of novel alleles. This study does not aim to serve as a comparison of the available software tools. The IMGT germline database version from January 2019 was used as a reference for allele annotation by IgBLAST.

We first analyzed the usage of all genes and the different alleles carried by individuals in the cohort. The relative usage of certain genes appeared to be strongly affected by the alleles present in the inferred genotype (Supplementary Figure S2). For example, when comparing IGHV1-2 homozygotes, individuals that were homozygous for allele *02 were generally found to have higher relative usage of this gene than those homozygous for allele *04. This was also true for certain alleles of IGHV1-46, IGHV3-11, IGHV3-43, IGHV3-48, IGHV3-53, IGHV4-61, and IGHV5-51. Overview of the usage of all genes that were detected in the inferred genotype of at least one individual can be found in Supplementary Figures S2-S3.

### Analysis of the V-REGION reveals 25 potential novel IGHV alleles

**Figure 2.**
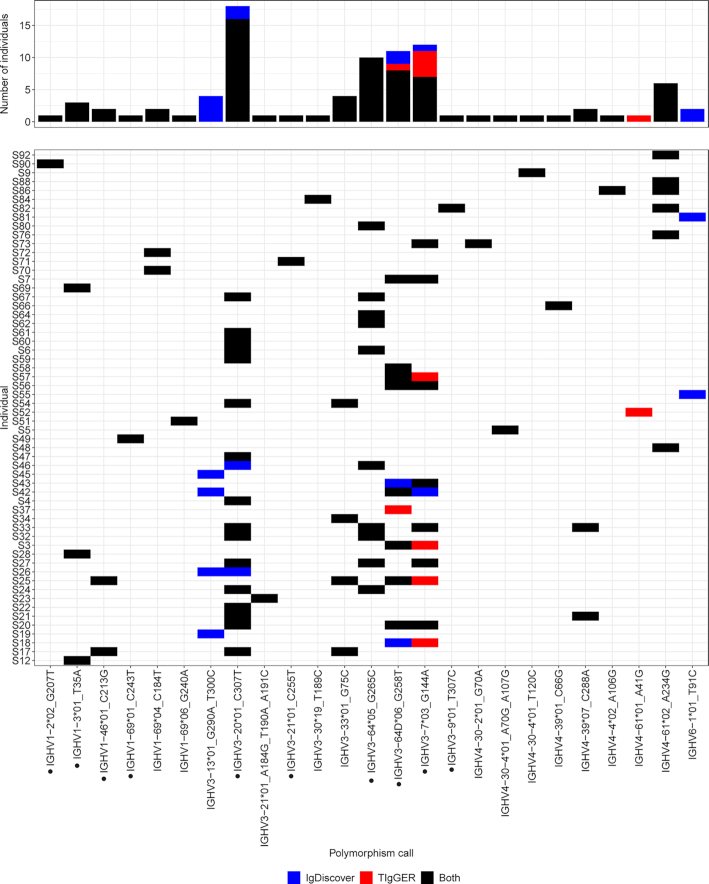
**Novel IGHV alleles**. The software suites TIgGER and IgDiscover were used to infer a personal IGHV genotype for each individual and to infer previously undiscovered alleles. All novel alleles that are part of a genotype inferred by at least one of the methods appear on the x-axis. Alleles that were validated by Sanger sequencing are marked with a dot. Individuals with at least one novel allele lie on the y-axis and are labeled by their subject name. For each allele, the color of a tile (or a bar) represents the method of detection and genotype inference. The height of each bar on top represents the number of individuals for whom a certain allele was inferred and is part of a genotype.

Although both TIgGER and IgDiscover can be used infer novel alleles from immunoglobulin repertoire-sequencing data, the type of data that each software suite was built to analyze is different. IgDiscover was made to analyze only naïve immunoglobulin repertoire data, while TIgGER can be used on clonally expanded repertoires as well. To make the settings as similar as possible, we adjusted the germline_min in TIgGER to 50, while keeping the IgDiscover parameters as default. To ensure that any potential software biases and PCR or sequencing errors were filtered out, we carefully inspected all candidates for novel alleles. Suspected false positive signals were filtered out from the novel allele candidates based on their mismatch frequency (Supplementary Figure S1) as described in the Methods section. Novel allele candidates that were determined to be false positives contained mutations A152G and T154G (found mainly by IgDiscover); and A85C (found mainly by TIgGER). In the end, we inferred altogether 25 potential novel alleles (Figure 2), and we named them using the closest reference allele. The majority of the novel alleles (22 out of 25) were identified both with TIgGER and IgDiscover. In addition to these, two novel alleles were found exclusively by IgDiscover, and one novel allele was found exclusively by TIgGER.

Thirteen novel alleles were selected for validation by targeted amplification and subsequent Sanger sequencing of gDNA (Supplementary Figure S4) of non-B cells, i.e.T cells and monocytes isolated by fluorescence-activated cell sorting (30). The validation primers are specified in Table S1. Out of those thirteen alleles, ten were successfully validated. These include IGHV1-2*02_G207T, IGHV1-3*01_T35A, IGHV1-46*01_C213G, IGHV1-69*01_C243T, IGHV3-7*03_G144A, IGHV3-9*01_T307C, IGHV3-20*01_C307T, IGHV3-21*01_C255T, IGHV3-64*05_G265C, and IGHV3-64D*06_G258T. Surprisingly, IGHV3-64*05_G265C was found to originate from IGHV3-64D (Figure 4C) and the obtained gDNA sequence was manually annotated as IGHV3-64*06D_C210A. During the course of our work, two of the novel alleles, namely IGHV1-46*01_C213G and IGHV3-20*01_C307T, were added to the IMGT database in July 2019 as IGHV1-46*04 and IGHV3-20*04 respectively.

Validation of the novel alleles revealed additional polymorphisms outside of the V-REGION. The allele IGHV3-64*06_G258T has a polymorphism in L-PART1 (position -21) in addition to the V-REGION polymorphism. Genomic validation of IGHV3-7*03_G144A revealed a further polymorphism in the intron. During validation of this allele, we also managed to amplify the genomic sequence of IGHV3-7*02, which carried the previously reported polymorphism A318G (44), which has recently been assigned as allele IGHV3-7*04. This polymorphism was not inferred from the AIRR-seq data in our study, since the default parameters of the inference tools are set to detect polymorphisms up to position 312.

Attempts to validate IGHV4-39*07_C288A, IGHV4-61*02_A234G, and IGHV6-1*01_T91C were unsuccessful. The gene-specific primers that were used for validation were designed based on the current reference genome. However, the efficiency of the IGHV4 primers was inferior, and Sanger sequencing only revealed allele *01 of each gene, even in clearly heterozygous individuals.

### Analysis of upstream sequences yields a more complete and accurate germline reference dataset

As some of the validated novel alleles had additional polymorphisms in the intron or the leader sequence, we extended our analysis of the AIRR-seq data beyond the V-REGION. Although introns are not present in the AIRR-seq data, the sequences of the 5′ untranslated region (5′UTR), L-PART1, and L-PART2 lie upstream of the V-REGION and are present in the data thanks to the library preparation method (Figure 1). We will refer to 5′UTR, L-PART1, and L-PART2 collectively as upstream sequences.

**Figure 3.**
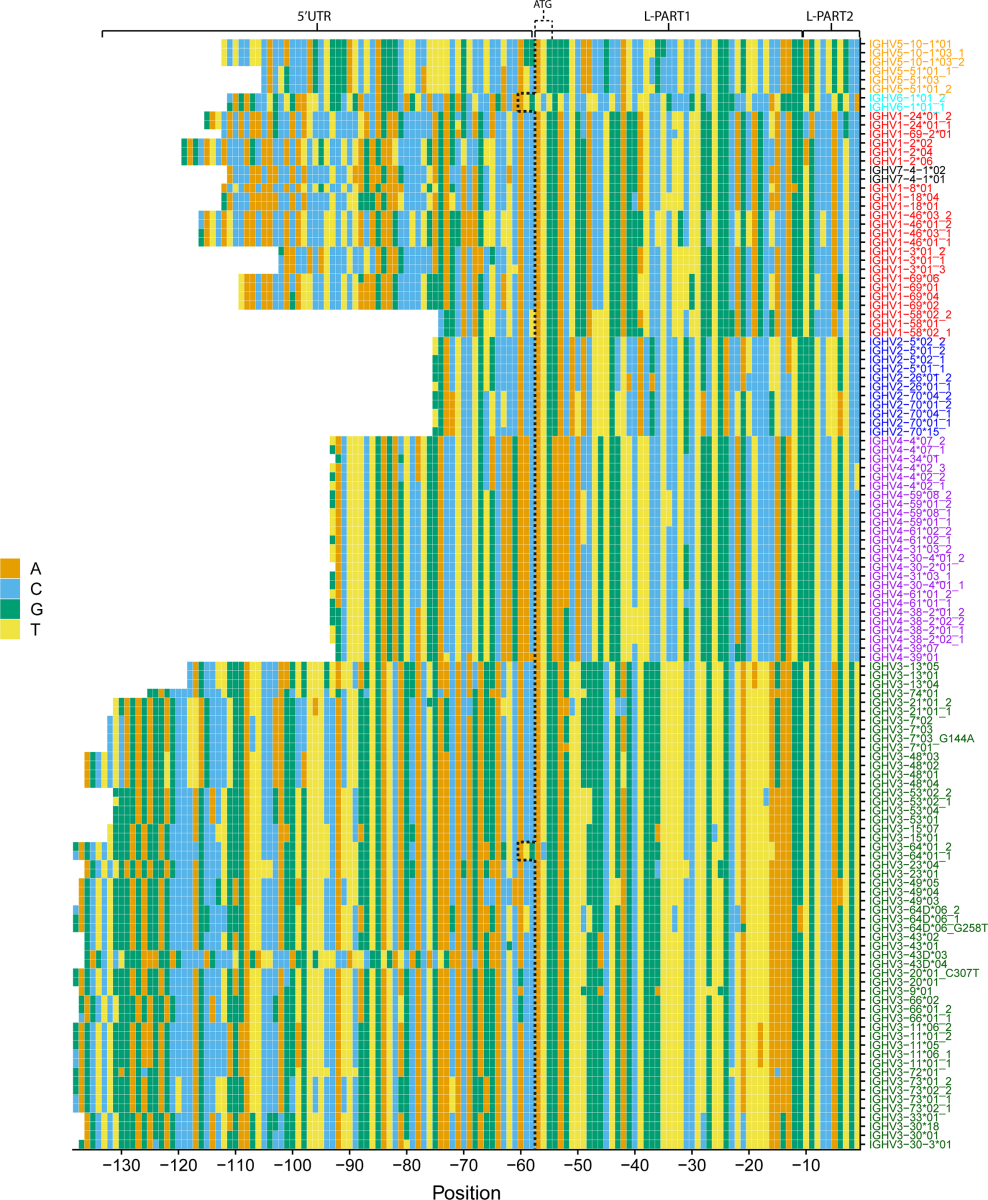
**Upstream germline dataset**. For each allele, consensus upstream sequences were built. Consensus sequences constructed from clusters with less than 10 sequences or with relative frequency <0.1 were excluded. Each row represents a consensus upstream sequence of a V allele with 5′ to 3′ orientation. The colors of the tiles represent the different nucleotides. The coordinates on the x-axis describe the position of each nucleotide relative to the start of the V-REGION (5′ to 3′) and are therefore labelled as negative numbers. Alleles with more than one consensus sequence are marked with the allele name followed by an underscore and the respective consensus sequence number. For example, the two different consensus sequences for allele IGHV3-64*01 are marked as IGHV3-64*01_1 and IGHV3-64*01_2. The number of individuals who carry each variant is shown in Supplementary Figure S5.

We decided to use the genotyped AIRR-seq data to characterize upstream sequence variants for all genes and alleles. To extract the upstream sequences, we removed the VDJ and constant regions, while keeping the original sequence's V-REGION annotation. Sequences from each individual were processed separately. We observed slight variations in the length of 5′UTRs assigned to the same gene. It is important to have matching length for clustering, as different lengths could mean that identical sequences would not cluster together. To overcome this issue, for each gene we trimmed the ends of 5′ ends of the upstream sequences to match the most frequent length. We then took the trimmed upstream sequences with the same allele annotation and clustered them. Each cluster of a sufficient size gave rise to one consensus upstream sequence. This process was repeated for all genes and alleles across all individuals. Finally, consensus sequences from all individuals were combined to create an upstream germline reference dataset of the cohort (Figure 3). The number of individuals carrying each of the variants is shown in Supplementary Figure S5.

According to the constructed germline reference dataset, the lengths of L-PART1 (45 nt) and L-PART2 (10 nt) sequences appear to be well conserved across most genes, with the exception of IGHV3-64*01 and IGHV6-1*01 (Figure 3). The L-PART1 sequences of these two genes are 3 nt longer, which makes the position of ATG appear to be shifted upstream. The length of the 5′UTR is relatively conserved within the same gene family, however, there is a large variability across different families. Genes of the IGHV2 family have the shortest 5′UTR, while the 5′UTRs of IGHV3 genes are the longest.

Comparison of the consensus sequences in the cohort with the reference sequences obtained from the IMGT/GENE-DB (11) revealed some discrepancies between our data and the reference database. For example, the IMGT reference sequence of the allele IGHV5-51*01 has T at position –3 in L-PART2, while the reference sequences of the other reference alleles have G at this position. However, in our data, all IGHV5-51 alleles have G at position –3, as illustrated in Figure 3. Our observation of G at position –3 in IGHV5-51*01 was validated by targeted amplification and Sanger-sequencing of IGHV5-51*01 from a homozygous individual (Supplementary Figure S6).

### Length of 5′UTRs correlates with the distance between TATA-box and start codon

As depicted in Figure 3, the length of the 5′UTR differs between IGHV gene families, but is relatively conserved within a gene family. To investigate whether the different length of 5′UTRs among the different families had any correlation with the distance from the promoter elements, we decided to inspect the reference gDNA sequence from the IMGT database. We collected the available germline reference sequences of the upstream flanking regions of V-gene promoters from the IMGT/GENE-DB and aligned them to look for conserved patterns.

Using the sequences from the IMGT reference database, we determined the distance between the ATG start codon and the reference or putative TATA-box. We found that this distance varied greatly between different gene families. By comparing this distance to the 5′UTR length from the AIRR-seq data, we observed that the distance between the ATG and the TATA-box correlated with the length of the 5′UTR (Supplementary Figure S7). Sequences with longer ATG to TATA-box distance had longer 5′UTRs.

### Differences in the upstream sequences can aid allele annotation

**Figure 4.**
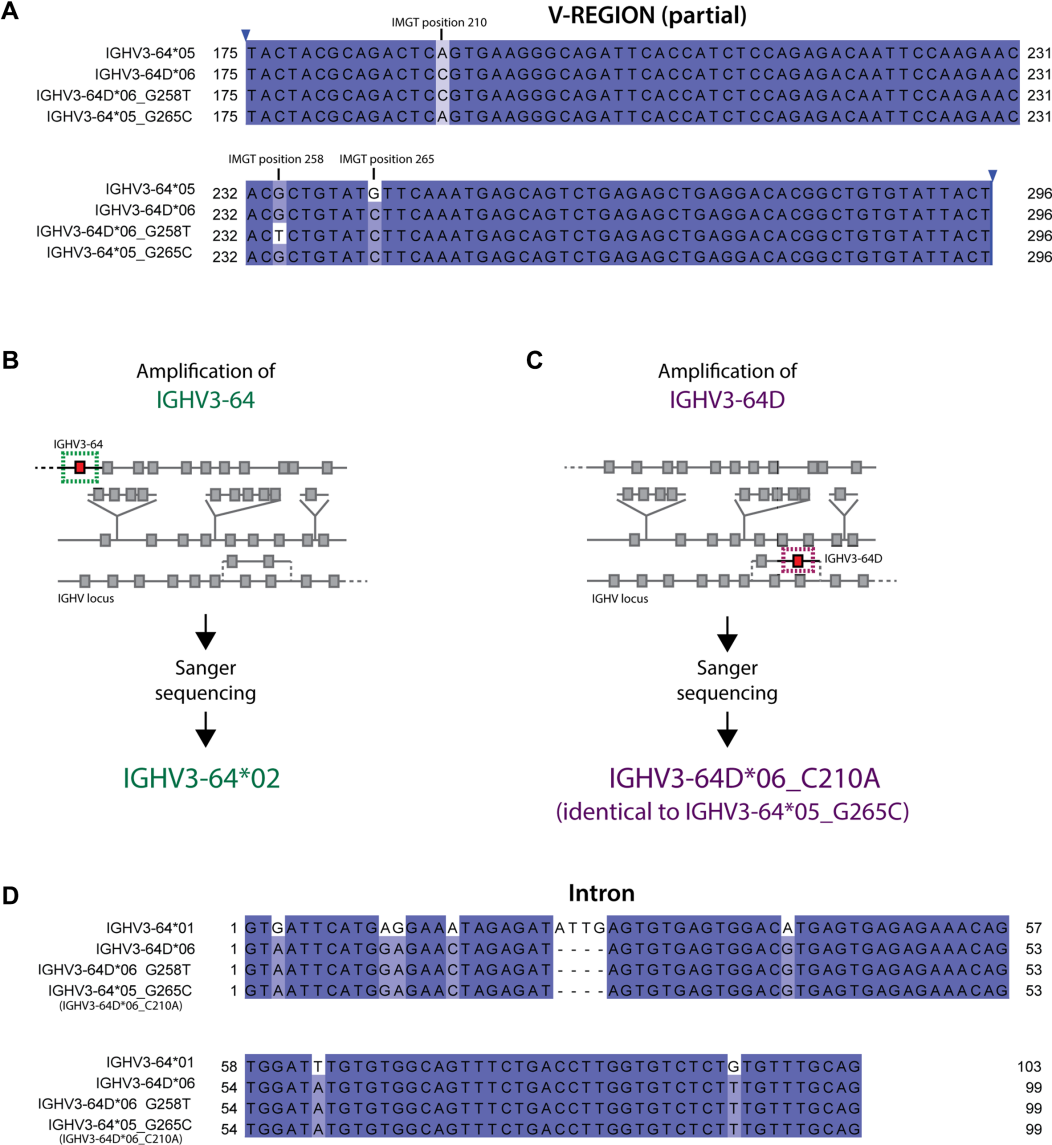
**Genomic validation of** **IGHV3-64/IGHV3-64D alleles**. (**A**) The alleles IGHV3-64*05 and IGHV3-64D*06 differ in only two positions within the V-REGION. A novel allele identified from our AIRR-seq data, IGHV3-64*05_G265C, differs from each of them only by 1nt. Although annotated by IgBLAST as IGHV3–64, its upstream region matched IGHV3-64D better (not shown). To validate this novel allele and ensure its correct annotation, we PCR amplified the genes IGHV3-64 and IGHV3-64D from gDNA of a carrier of this novel allele using gene-specific primers. (**B**, **C**) The process of validation of IGHV3-64*05_G265C depicted with a schematic IGHV locus representation. The novel allele was originally assigned as being closest to IGHV3-64*05, however, this allele was not amplified by primers specific for IGHV3-64. The novel allele IGHV3-64*05_G265C was only detected when IGHV3-64D was amplified. Since the annotation software incorrectly aligned it to IGHV3–64*05, it was manually annotated as IGHV3-64D*06_C210A. Despite the different names, both IGHV3-64*05_G265C and IGHV3-64D*06_C210A refer to exactly the same sequence, the former is IgBLAST annotation and the latter is manual annotation. (**D**) Comparison of the intronic regions of IGHV3-64*01, IGHV3-64D*06 and the novel IGHV3-64D alleles. The reference sequence of IGHV3-64*05 in the IMGT database is partial and lacking the intron, and therefore could not be compared. Instead, the intron of IGHV3-64*01 is shown. The intron of the novel allele originally annotated as IGHV3-64*05 G265C matches the one of IGHV3-64D. The numbers in the alignments (**A**, **D**) do not follow the unique IMGT numbering, however, for the polymorphic positions in the V-REGION (**A**) their unique IMGT numbers are shown above the alignment.

The novel allele IGHV3-64*05_G265C was initially not validated by amplification of the gene IGHV3-64, as Sanger sequencing revealed only IGHV3-64*02 in a selected individual carrying the suspected polymorphism, and with no sequence corresponding to allele *05 being present (Figure 4B). Originally, this allele was ambiguously annotated as deriving from either IGHV3-64*05 or IGHV3-64D*06, as it differs by one nucleotide from each of these alleles (Figure 4A).

The upstream sequences of IGHV3-64 and IGHV3-64D differ across their entire length, including the 5′UTR, L-PART1 and L-PART2 (Figure 3). The upstream regions of the novel allele IGHV3-64*05_G265C are identical to those of IGHV3-64D, which indicated that this is indeed an allele of IGHV3-64D and not IGHV3-64. Therefore, we decided to amplify the gene IGHV3-64D using primers specific to the duplicated gene only. This resulted in the novel allele being finally validated (Figure 4C). Upon obtaining the full germline sequence of the novel allele, we observed that its intron matched the one of IGHV3-64D and not IGHV3-64 (Figure 4D).

The genes IGHV3-43 and IGHV3-43D are another example of duplicated genes with differences in the upstream sequences. Unlike the previous example, IGHV3-43 and IGHV3-43D seem to have identical L-PART1 and L-PART2 sequences but differ in the 5′UTR (Figure 3). However, not only genes, but also some alleles of the same gene can be distinguished by their upstream sequences. The novel allele IGHV3-64D*06_G258T differs from IGHV3-64D*06 in one position located in L-PART1. Similarly, IGHV4-39*01 and IGHV4-39*07 have three differences within the 5′UTR; and the alleles IGHV3-43*01 and *02 differ in one position within the 5′UTR.

## DISCUSSION

Our analysis of the naïve B cell immunoglobulin repertoire data from 98 individuals revealed several novel polymorphisms both in the coding and in the upstream sequences of IGHV genes. To our knowledge, we are the first to provide a comprehensive overview of the heavy chain upstream (5′UTR, L-PART1, and L-PART2) sequence variants in an AIRR-seq dataset. We managed to validate a number of novel alleles by targeted amplification of genomic DNA of the same individuals. In addition, we provide further genomic evidence of IGHV3-7*04, which differs from IGHV3-7*02 at position 318 (31,44) and has been recently added to the IMGT germline reference database (November 2019).

We faced several issues with missing or incomplete genomic reference sequences, which complicated the design of efficient primers for verification of novel alleles. Some of our validation attempts were unsuccessful resulting only in the amplification of a ‘wild-type’ allele without a polymorphism. We suspect this might be caused by allelic dropout (45,46). As we show in our upstream sequence overview (Figure 3), alleles IGHV4-39*01 and IGHV4-39*07 differ at multiple positions within the 5′UTRs. Our primers were designed to bind flanking sequences of the gene, and their design was based on the current reference genome, which contains the allele *01 of IGHV4-39. Potential differences in the primer binding regions could be the cause of a failure to amplify the novel alleles, in this case IGHV4-39*07_C288A.

Although AIRR-seq studies are very useful for characterizing variation in immunoglobulin genes, one of the main limitations are issues with gene and allele annotation (47). The V-REGION is annotated based on the most similar allele in the reference database. However, since the V genes are highly similar, this annotation might not always be correct. Incorrect gene assignment could lead to potential downstream errors in analysis. In our study, the novel allele originally annotated as IGHV3-64*05_G265C was later found to be derived from the gene IGHV3-64D, located on a different part of the IGHV locus than IGHV3-64. As previously shown (5,6,10), IGHV3-64D is likely a part of an alternative haplotype, since it was found to be deleted in many individuals, even in this cohort (30). These two genes differ in their upstream sequences, and thanks to this distinction, we were able to correctly assign the novel allele to IGHV3-64D and validate it from gDNA.

Our results demonstrate that polymorphisms in the upstream regions can be utilized to improve annotation methods presently employed. Having said that, the genetic variation in the sequences upstream of the V-REGION is currently poorly characterized. Many reference sequences, which were deposited to the IMGT germline database are partial and contain only the V-REGION sequence. The V-REGION has long been the main focus of studies looking at IGHV genes, since mutations in this part of the gene can have functional effect on the resulting protein. Even for validation purposes, historically it was only the V-REGION that was amplified. However, as we have shown here, upstream sequences can be of a great value, and it is good to pair AIRR-seq data analysis with targeted gDNA sequencing to also capture introns that can reveal more about the genomic location. It is surprising that the genetic variation in the upstream regions is still overlooked, considering the fact that the leader regions (L-PART1, L-PART2) are nowadays frequently used as primer binding sites for immunoglobulin repertoire library preparation protocols (31,48,49).

The reason for the existence of upstream polymorphisms is unclear, but conceivably such polymorphisms might have functional relevance by influencing the stability of the mRNA or by affecting the binding of regulatory proteins (50,51). Further studies are needed to explore polymorphisms in the upstream sequences and to determine whether they have any functional effect. Association of these allelic variants with disease can be studied in sufficiently powered studies. In addition, more genomic studies could be performed to characterize their promoters and other regulatory elements, which might help explain the differences in expression levels across individuals.

## DATA AVAILABILITY

The pipeline for novel allele discovery and genotype processing using the software tools TIgGER and IgBLAST is available on the VDJbase website (https://www.vdjbase.org). Custom code for the analysis of upstream sequences is available at https://bitbucket.org/yaarilab/cluster_5utr/src/master/. Sanger sequences of validated IGHV alleles have been deposited in the GenBank under accession numbers: MN337615 (IGHV1-2*02_G207T), MN337616 (IGHV1-3*01_T35A), MN337617 (IGHV1-46*01_C213G), MN337618 (IGHV1-69*01_C243T), MN337619 (IGHV3-7_G144A_T300C), MN337620 (IGHV3-7*02_A318G/IGHV3-7*04), MN337622 (IGHV3-20*01_C307T), MN337623 (IGHV3-21*01_C255T), MN337624 (IGHV3-64D*06_G258T), MN337625 (IGHV3-64D*06_C210A), and MT078123 (IGHV5-51*01). The upstream sequence variants are provided as a supplementary material in the form of an Excel sheet.

## Supplementary Material

gkaa310_Supplemental_Files
