# Supplementary material for: Polymorphisms in human immunoglobulin heavy chain variable genes and their upstream regions
Source: Nucleic Acids Res. 2020 May 4;48(10):5499–510. doi: 10.1093/nar/gkaa310 (PMC7261178; doi:10.1093/nar/gkaa310)
Supplement: gkaa310_Supplemental_Files [file gkaa310_supplemental_files.zip › Mikocziova_et_al_Supplementary_data.pdf]

Polymorphisms in human immunoglobulin heavy chain variable genes and their upstream regions

Ivana Mikocziova[1†*], Moriah Gidoni[2†], Ida Lindeman[1], Ayelet Peres[2], Omri Snir[1], Gur Yaari[2‡], Ludvig M. Sollid[1‡]

[1] K.G.Jebsen Centre for Celiac Disease Research and Department of Immunology, University of Oslo and Oslo University Hospital, 0372 Oslo, Norway
[2] Faculty of Engineering, Bar Ilan University, Ramat Gan 5290002, Israel

† Joint First Authors

‡ Joint Last Authors

* To whom correspondence should be addressed. Email: ivana.mikocziova@medisin.uio.no,
Correspondence may also be addressed to: l.m.sollid@medisin.uio.no

Supplementary Data

This document contains additional plots and figures that support the main data presented in the paper. In addition, two supplementary files are provided: a fasta file containing gDNA sequences from Sanger sequencing, and a table containing information about the upstream sequences (5'UTR, L-PART1 and L-PART2).

The supplementary .xls table contains the comparison of all detected upstream variants that passed the filtering steps with their available IMGT reference sequences obtained by searching the IMGT/GENE-DB. The first five columns represent the IMGT/GENE-DB reference: the reference allele name (REF_ALLELE) and the upstream IMGT reference sequences (REF_5'UTR, REF_L-PART1, REF_L-PART2, REF_FULL). Note that the 5'UTR, L-PART1 and L-PART2 are in 5'-3' orientation, while the REF_FULL is in 3'-5' orientation so that it can be compared with the 3'-5' CONSENSUS_SEQUENCE. FAMILY represents the IGHV family to which the gene (GENE) belongs to. CONSENSUS_SEQUENCE is the 3'-5' nucleotide sequence of an upstream variant detected by clustering, and the ALLELE_UPSTREAM is the variant name assigned to this consensus sequence. The different upstream variants for the same allele are distinguished by an underscore followed by a number. The CLUSTER_COUNT represents the number of individuals in which this upstream variant was identified. Variants detected in less than 10 individuals were filtered out. The FRACTION column shows what fraction of the total sequences assigned to the germline allele this upstream variant represents. For example, the IGHV1-58*02_1 variant was present in 47% of all sequences assigned to IGHV1-58*02 (FRACTION=0.468), while the IGHV1-58*02_2 variant was present in 53% of all IGHV1-58*02 sequences (FRACTION=0.532). The last two columns show the comparison between the upstream variant and its respective IMGT germline reference. The REF_MISSING column shows if any of the 5'UTR, L-PART1 or L-PART2 reference was missing from IMGT/GENE-DB. The value 0 means nothing was missing, while 3 means that all three parts were missing, i.e. there was no reference for the upstream sequences. The VARIATION column shows the mismatch between REF_FULL and CONSENSUS_SEQUENCE. The polymorphisms number is the nucleotide number within the 3'-5' sequence, and the letters represent the nucleotide change. MATCH means the CONSENSUS_SEQUENCE matches the REF_FULL; and NA means that comparison was not possible due to incomplete reference.

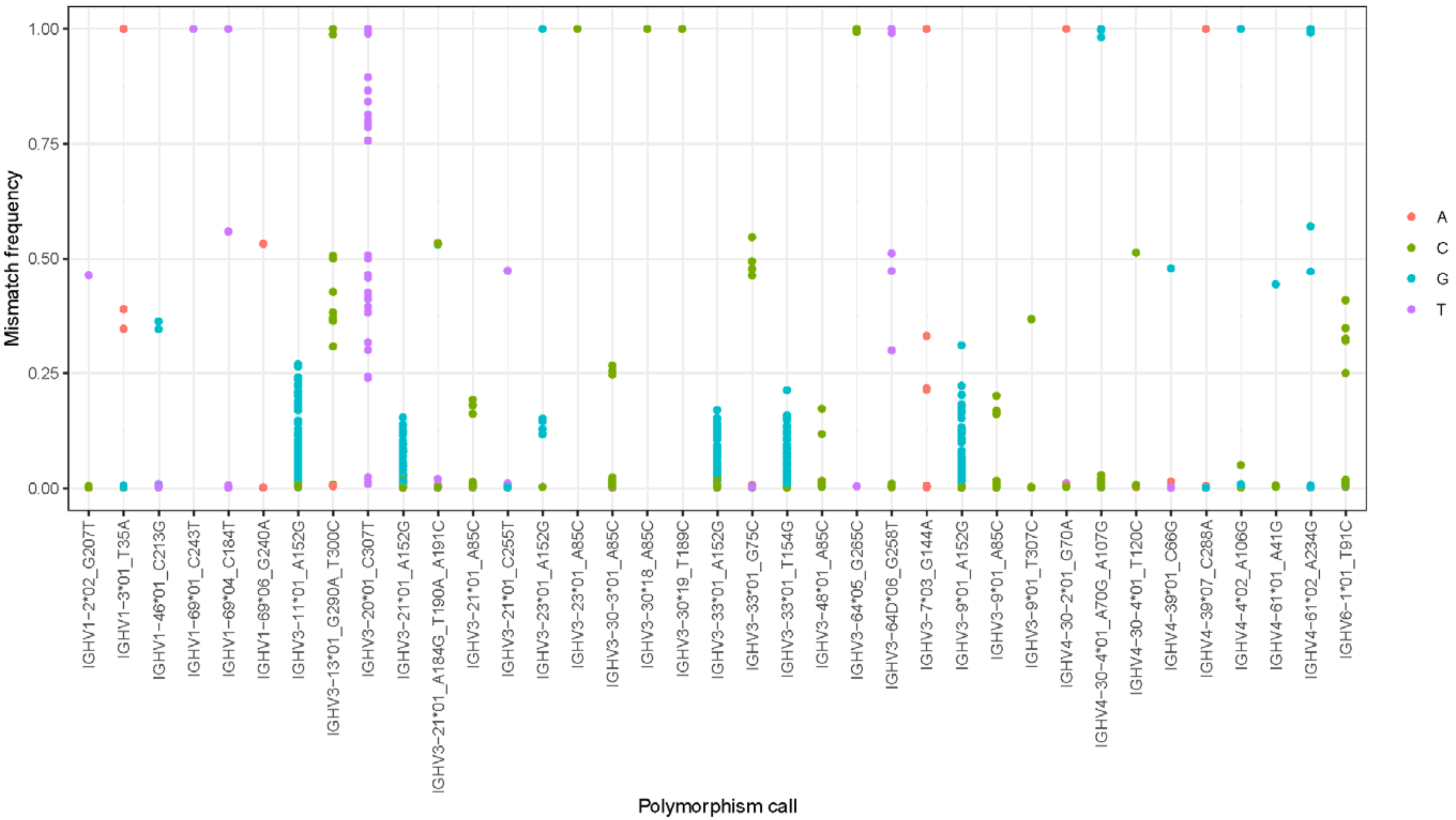

Figure S1. Germline mismatch frequency. To determine false positive candidates, we analysed the mismatch frequencies of novel allele candidates. For each individual, relative frequencies of polymorphisms (y-axis) were calculated for positions in sequences aligned to an allele, for which a novel allele candidate was inferred in the dataset (x-axis). Each dot represents a mismatch frequency for an individual for a certain allele and nucleotide. The color of the dot represents the nucleotide that does not match the germline. Novel allele candidates with mismatch frequency below 0.35 were considered false positives. More details about the filtering can be found in the Methods section.

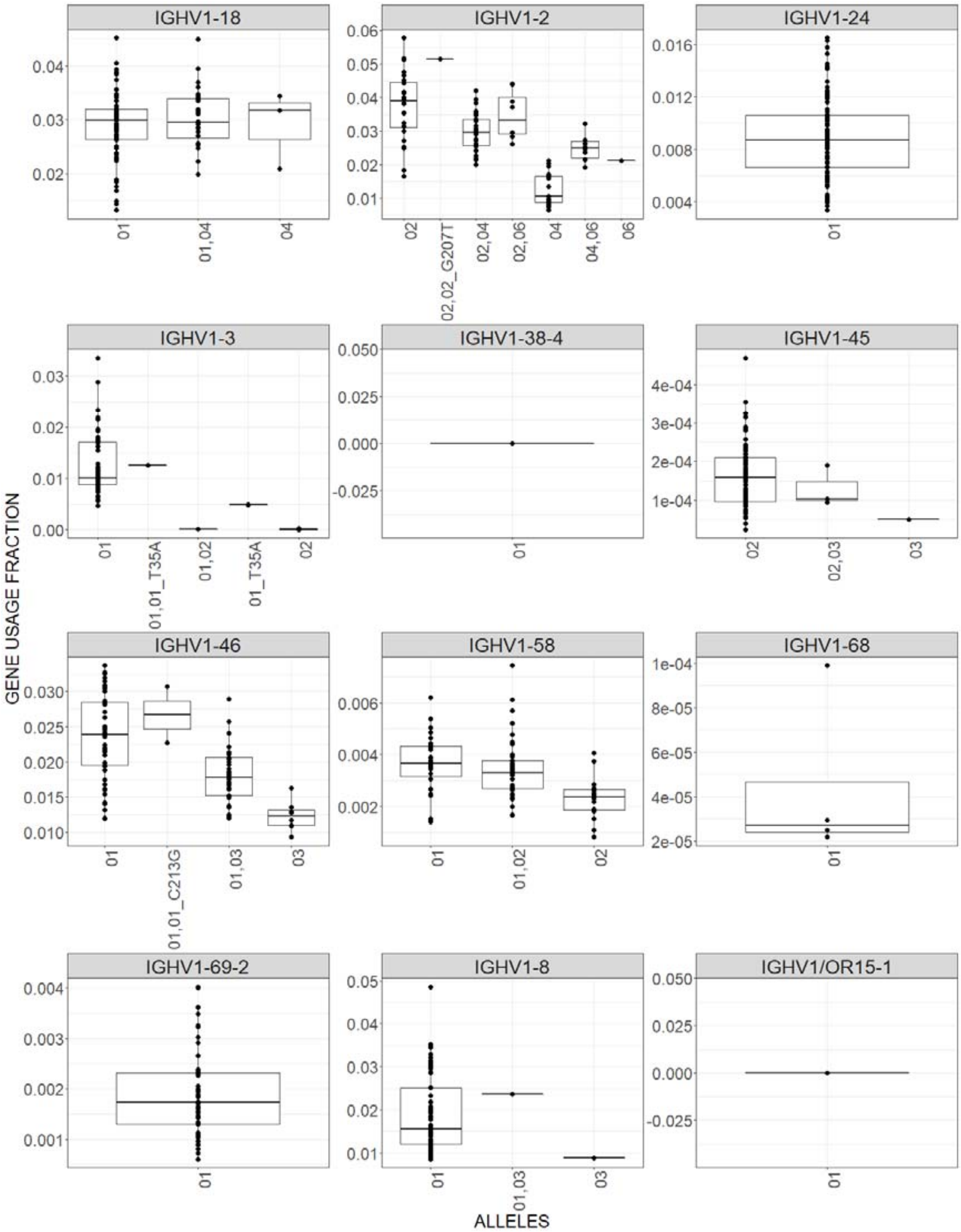

Figure S2. Usage of genes across individuals in the cohort. For each allele of a gene, we calculated its relative usage fraction in each individual. The usage fractions of alleles of the same gene in the same individual were then summed, revealing the gene's usage fraction. The x-axis shows the inferred allele, or multiple alleles, that were found in an individual's inferred genotype. Each dot represents one individual. The y-axis shows the relative usage fraction of a gene within the expressed repertoire. The bar represents the median value. A bias can be observed in some genes, where the median gene usage is higher in individuals homozygous for a specific allele than those homozygous for another allele. This figure continues on the next five pages.

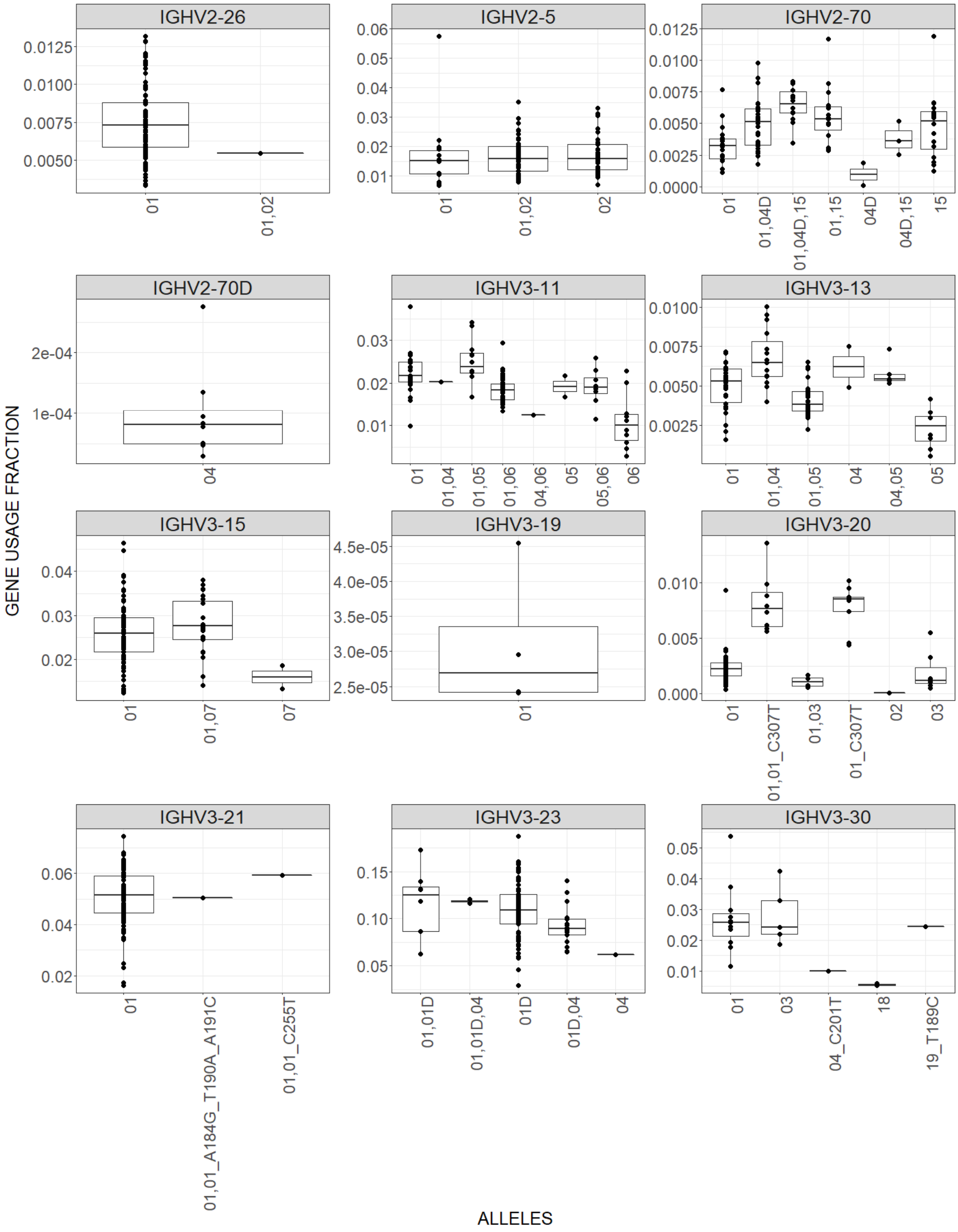

Figure S2. continued

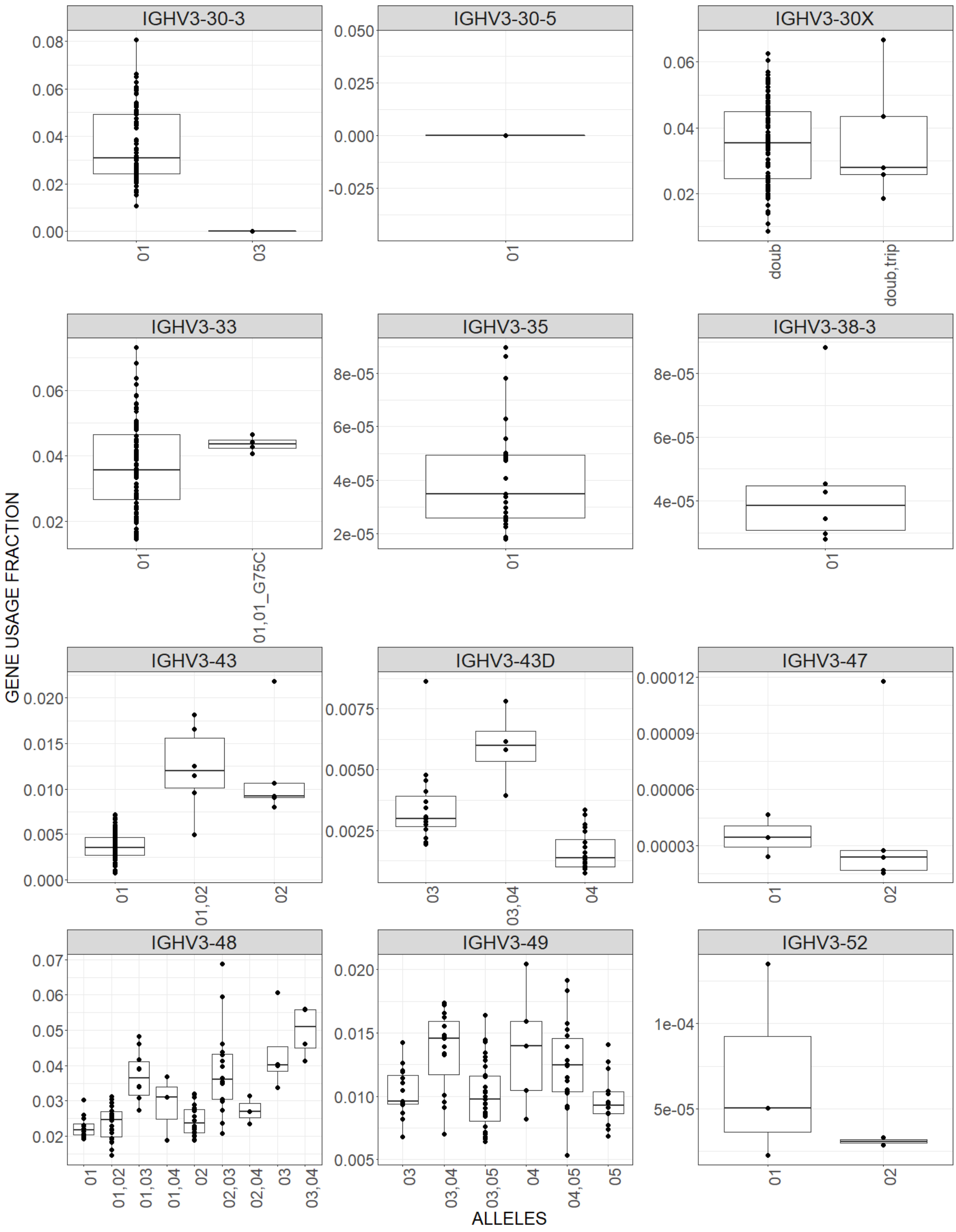

Figure S2. continued

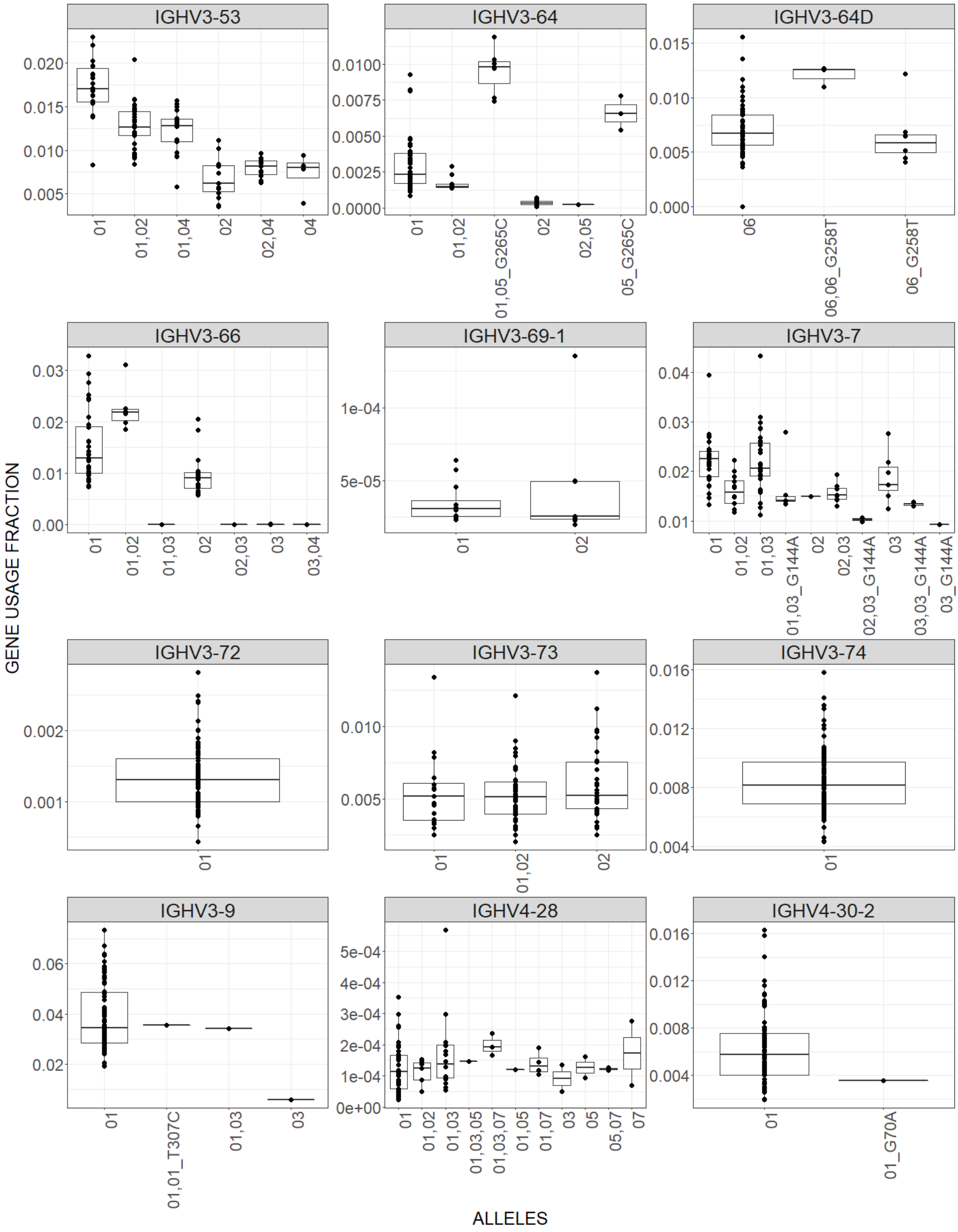

Figure S2. continued

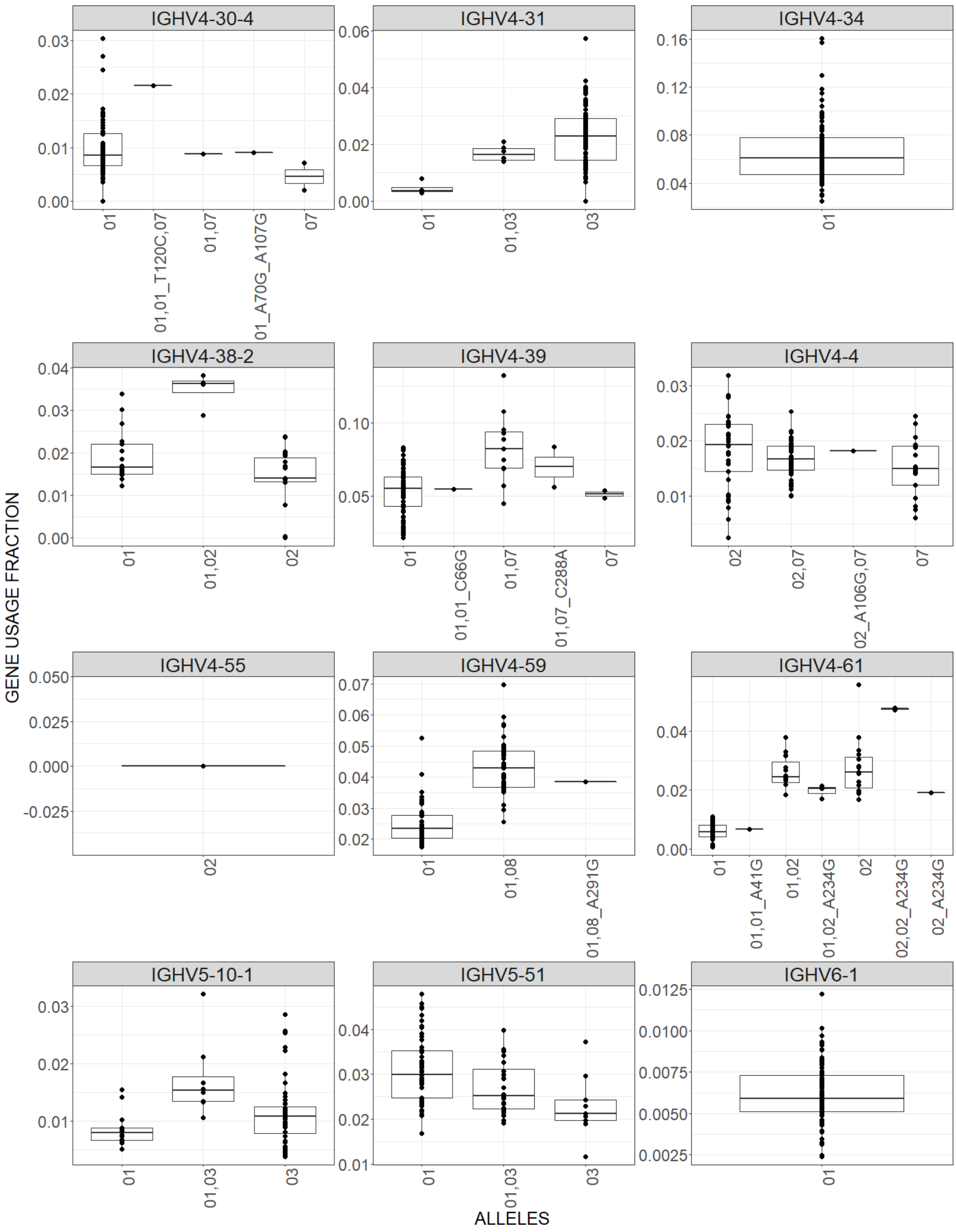

Figure S2. continued

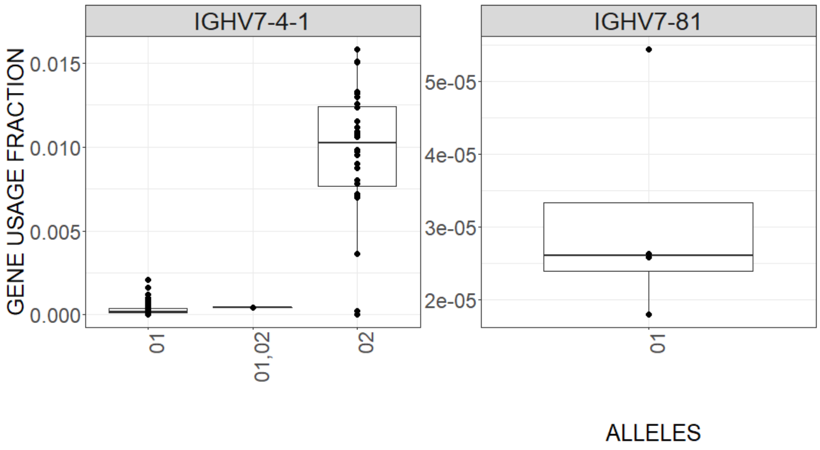

Figure S2. continued

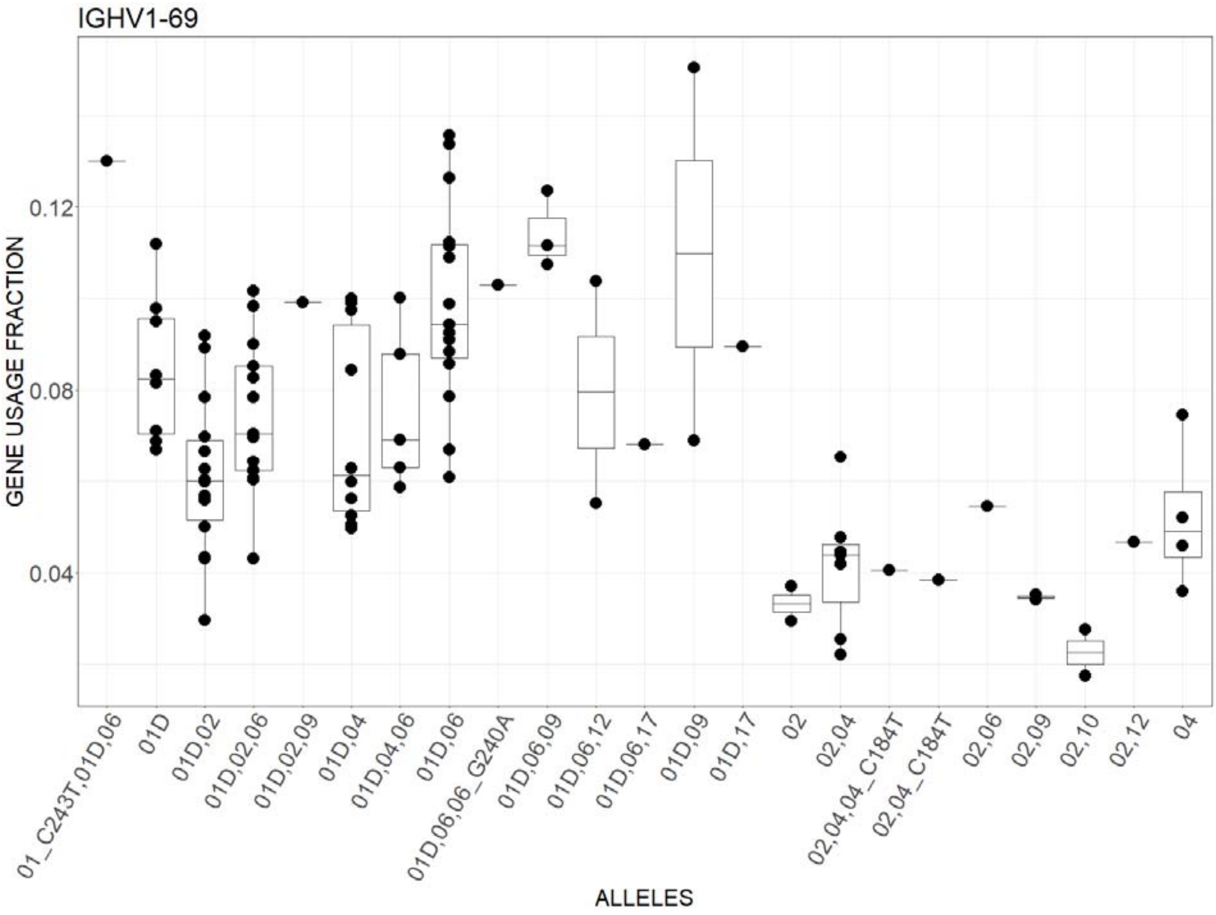

Figure S3. Usage of IGHV1-69 across individuals in the cohort. Relative usage fraction was calculated for each allele separately and in each individual, and the relative fractions of all expressed alleles were summed up. Different combinations of expressed alleles are shown on the x-axis, and the summed gene usage fraction is shown on the y-axis. Each dot represents one individual. The bar in the boxplot represents the median value.

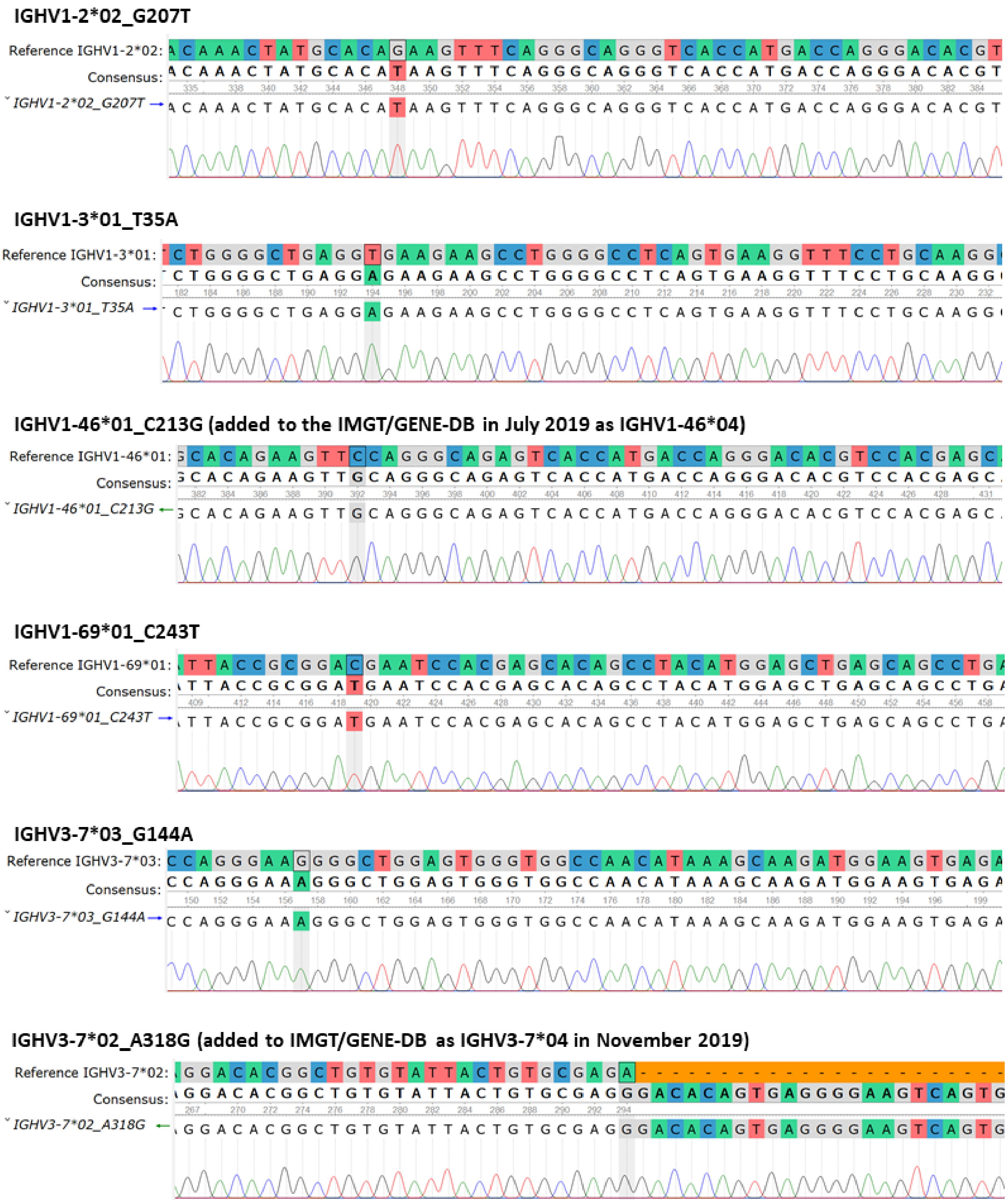

Figure S4. Sanger sequencing results. Ten novel alleles were validated by targeted amplification and subsequent Sanger sequencing. The trace files were aligned to reference sequences from IMGT GENE-DB (1) and visualised by UGENE (2). Despite its original IgBLAST annotation, the novel allele IGHV3-64*05_G265C was validated from IGHV3-64D. Its gDNA sequence was then manually annotated as IGHV3-64D*06_C210A. This figure continues on the next page.

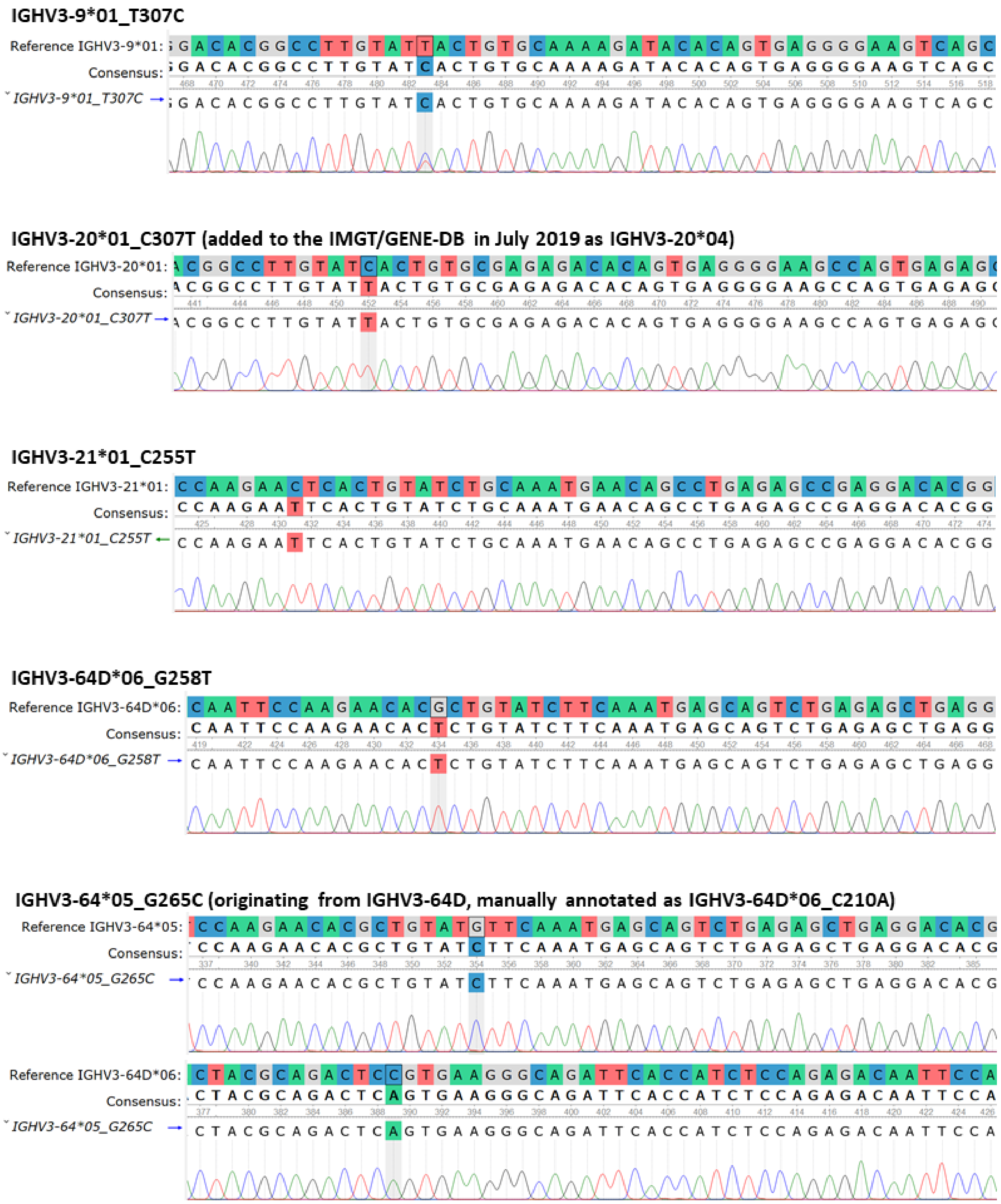

Figure S4. continued

Table S1. Primers used for genomic validation.

Primer name	Nt sequence (5‘-3‘)
IGHV1-2_fwd	CGGGAACTTGTTTTCAGCAGAC
IGHV1-2_rev	TTTCATTTCTCAGCCCCAGCA
IGHV1-3_fwd	TCCAGTGGGAGAAGCTCTGT
IGHV1-3_rev	GTCATTTCCTCCATGCCAGC
IGHV1-46_fwd	CTGTGTGGCAGATGGGACAT
IGHV1-46_rev	TACTGAGTGTGGCCTTTCCC
IGHV1-69_fwd	TGGGAGCACAGCTCATCA
IGHV1-69_rev	CACTCTCAGGATGTGGGTTT
IGHV3-9_fwd	AGGACTCACCATGGAGTTGG
IGHV3-9_rev	TTTTTGTCTGGGCTCTCGCT
IGHV3-11_fwd	CAGCGTCCCACTAGAGCTTG
IGHV3-11_rev	CTGCAGGGAGGTTTGTGTCT
IGHV3-23_fwd	ATGCAAATAGAGCCCTCCGTCT
IGHV3-23_rev	TTCTGTCCCAGGACTGATTGCG
IGHV3-64_fwd	TTTGGGCTGAGCTGGGTTTT
IGHV3-64_rev	CAGGGAGGTTTCTGCATGGT
IGHV3-64D_fwd	AAGGACACTCTCATCTGCCC
IGHV3-64D_rev	CTCCTTGTGCACCTGCCTC
IGHV5-51_fwd	GAGAGGGACAATAGCAGGGTGTA
IGHV5-51_rev	CATATTGGAGAGGTGCCTGTTAGG
IGHV6-1_fwd	AGTCACCAGAGCTCCAGACA
IGHV6-1_rev	GCTCACACTGACTTCCCCTC
**Primers from Vázquez Bernat *et al.* (3):**	
IGHV3-7R	CCTGGGGAAATTTGACGACGAGGCA
IGHV3-7F	GGGTACAGCCTATTCCTCCAGCA
IGHV3-20R	GCACCTGGTCCCTGAGTTTACTGTGTTC
IGHV3-20F	CACGGGCCAGACAGTGAGACTGG
IGHV3-21R	CGCCGCAGGCCATGACAGGAAGC
IGHV3-21F	CAGCGTCCCACCCTAGAGCTTGT

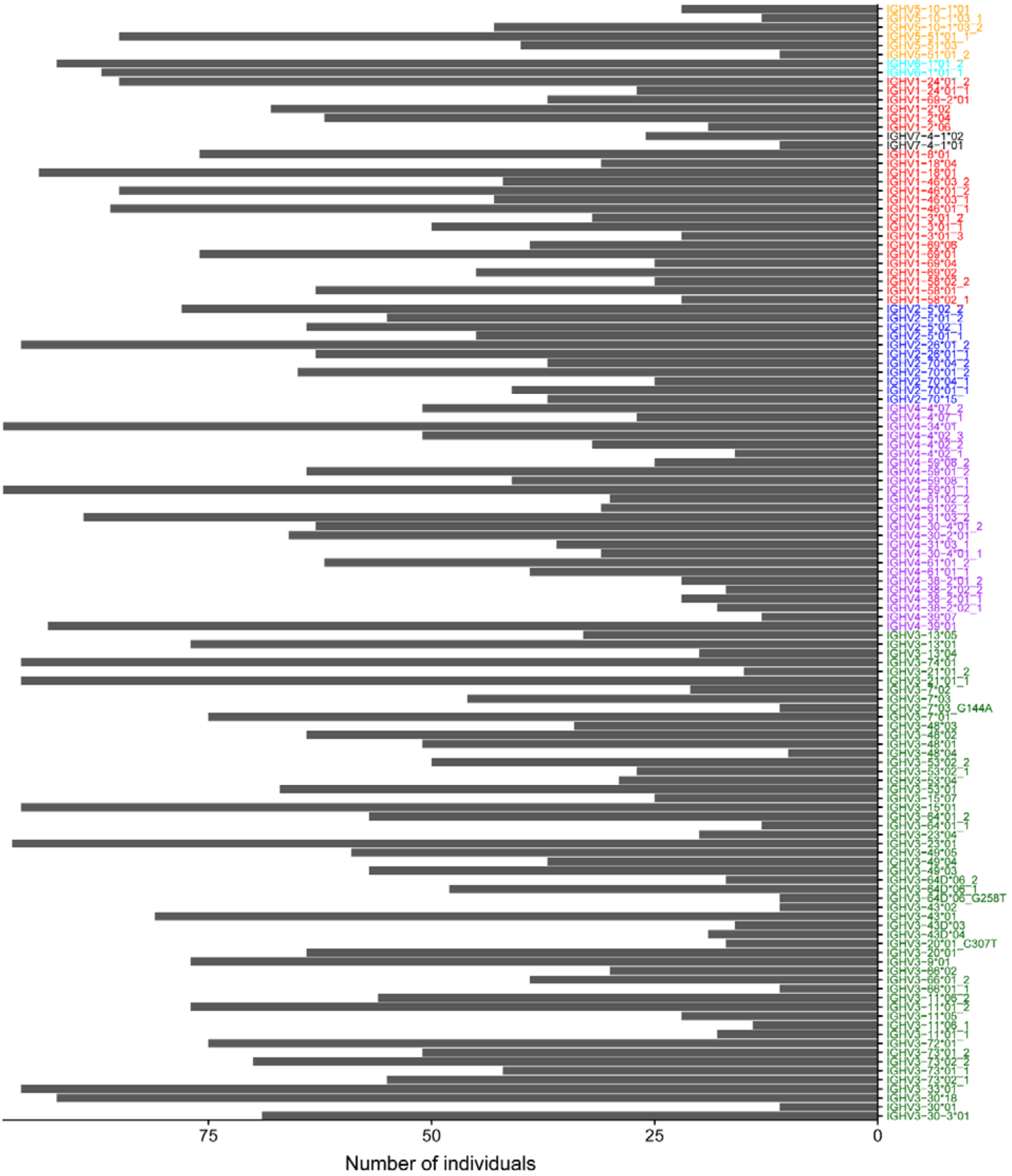

Figure S5. Number of individuals carrying the consensus 5'UTR sequence. Consensus 5'UTR sequences for each IGHV allele across all individuals were gathered and clustered to create a 5'UTR database. The length of each bar (x-axis) is the cluster size for a specific 5'UTR allele (y-axis), i.e. the number of individuals carrying the variant.

a

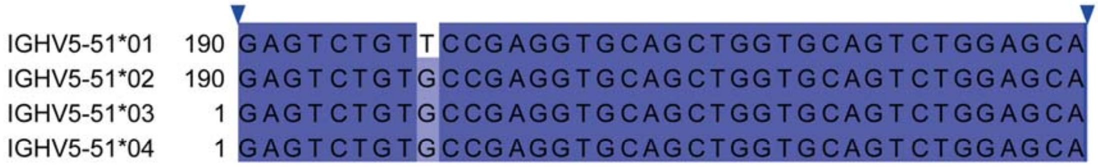

b

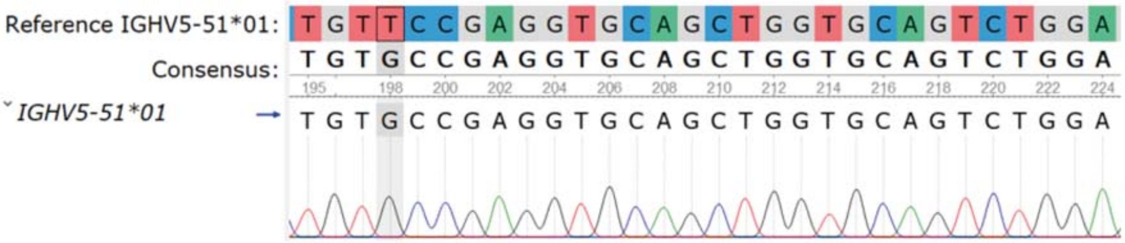

Figure S6. Validation of the IGHV5-51*01 L-PART2. The L-PART2 part of IGHV5-51*01 in all individuals in our cohort differed from the reference in the IMGT database. (a) Alignment of the reference L-PART2 sequences of selected IGHV5-51 alleles as obtained from IMGT/GENE-DB. (b) An individual from our cohort homozygous for IGHV5-51*01 was selected and IGHV5-51 was amplified using gene-specific primers (shown in Table S1). Sanger sequencing of the amplified product revealed that the L-PART2 of IGHV5-51*01 indeed differs from the reference.

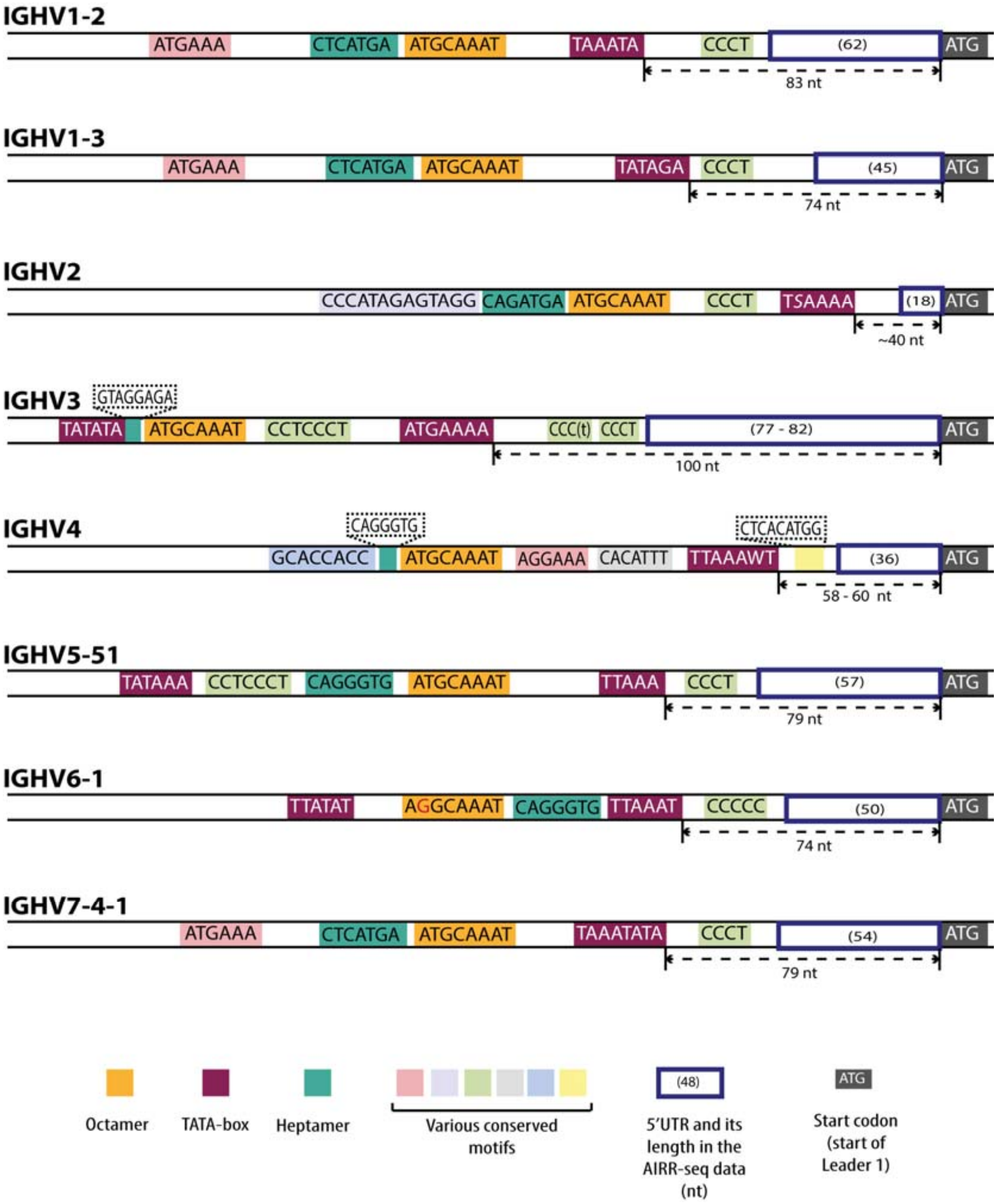

Figure S7. Schematic representation of the IGHV promoter regions. Reference upstream genomic sequences, including the promoter region were retrieved from the IMGT germline database and schematically depicted. Conserved motifs were identified by aligning all available 5'UTR and promoter reference sequences (> 150 nt) by MUSCLE and by searching for regions with high levels of homology. TATA-box sequences (in maroon) of some genes have been previously reported. For the remaining genes, we identified a putative TATA-box by searching for a TA-rich sequence. The octamer (in yellow) is well characterized and highly conserved across all genes. The heptamer (in dark turquoise) was only characterized for IGHV1 genes. In the other genes, we identified putative heptamers by searching for a conserved sequence upstream of the octamer. Various conserved motifs with unknown function were also identified (pastel colors). The ATG start codon is shown in grey. The 5'UTRs that are found in the AIRR-seq data are lined in dark blue, and their typical length in the repertoire sequencing data is shown in brackets. The length of the 5'UTRs correlated with the distance between the ATG and the TATA-box.

SUPPLEMENTARY REFERENCES:

1. Giudicelli, V., Chaume, D. and Lefranc, M.-P. (2005) IMGT/GENE-DB: a comprehensive database for human and mouse immunoglobulin and T cell receptor genes. *Nucleic Acids Research*, **33**, D256-D261.
2. Okonechnikov, K., Golosova, O., Fursov, M. and the, U.t. (2012) Unipro UGENE: a unified bioinformatics toolkit. *Bioinformatics*, **28**, 1166-1167.
3. Vázquez Bernat, N., Corcoran, M., Hardt, U., Kaduk, M., Phad, G.E., Martin, M. and Karlsson Hedestam, G.B. (2019) High-quality library preparation for NGS-based immunoglobulin germline gene inference and repertoire expression analysis. *Frontiers in Immunology*, **10**.
